# Comparison of framingham risk model, atherosclerotic cardiovascular disease risk model, and assign risk model in detecting sub-clinical atherosclerosis among DIMAMO residents, Limpopo province, South Africa

**DOI:** 10.3389/fcvm.2026.1726722

**Published:** 2026-02-19

**Authors:** Dinah Mohlele, Cairo Bruce Ntimana, Kagiso Peace Seakamela, Solomon S. R. Choma, Tumelo Satekge, Matimba Ringane

**Affiliations:** 1 Department of Pathology, University of Limpopo, Polokwane, South Africa; 2 DIMAMO Population Health Research Centre, University of Limpopo, Polokwane, South Africa; 3 Department of Pathology, University of Limpopo, Polokwane, South Africa

**Keywords:** association, cardiovascular disease, carotid intima media thickness, risk model, risk score

## Abstract

**Background:**

Metabolic and Cardiovascular risk factors affect the outcome of an individual's cardiovascular risk scores. There are several cardiovascular disease (CVD) risk models developed to predict CVD risk in individuals, although most of the CVD risk models are not validated, or their performance is understudied in some populations. The study aims to evaluate the performance ability of these CVD risk models in distinguishing individuals with increased Carotid Intima Media Thickness (CIMT).

**Methods:**

The study was retrospective and involved 245 participants' data. Three CVD risk models were evaluated and compared for the ability to determine the association between baseline risk scores and a cross-sectional marker of subclinical atherosclerosis. The data were analyzed using the Statistical Package for SPSS, version 30. A T-test was used to compare continuous CVD risk variables between groups, a Chi-square was used to compare the proportion of categories for the Framingham risk scores, the Atherosclerotic, and chi-square test was used to compare the proportion of categories for the Framingham risk scores, the Atherosclerotic Cardiovascular Disease (ASCVD) risk score, and the ASSIGN risk scores between groups. Logistic regression and Receiver Operating Characteristic (ROC) analyses were used to determine the model's accuracy in terms of sensitivity and specificity. A *p*-value of less than 0.05 was considered statistically significant.

**Results:**

The mean age for individuals with high risk was 60 years. The proportion of high Framingham risk score (FRS), intermediate ASCVD, high ASCVD, very high ASCVD, and high ASSIGN risk scores were statistically not significant between normal CIMT and increased CIMT participants. The Framingham risk model, the ASCVD risk model, and the ASSIGN risk model all showed no significant association with CIMT.

**Conclusion:**

In this study, the CVD risk models' performance and association were poor, with poor discriminative ability to distinguish individuals with increased CIMT.

## 1 Introduction

Worldwide, there is an increasing prevalence of non-communicable diseases (NCDs), with low- and middle-income countries being the most affected (1). Cardiovascular disease (CVD) is one of the leading causes of mortality, with the main prevalent cardiovascular diseases being stroke (2) and heart disease (3). There are various CVD risk models [the Framingham risk model, the Atherosclerotic Cardiovascular Disease (ASCVD) risk model, and the ASSIGN risk model] used to predict the risk of CVD events (4, 5). These models are mostly used in research to estimate the likelihood of future CVD events based on traditional risk factors such as age, sex, blood pressure, cholesterol levels, diabetes, and smoking status (6).

However, these CVD risk models can overestimate, especially the Framingham risk model, and underestimate, especially the ASCVD model risk, depending on the population and country-specific characteristics (7). While some CVD risk models have been documented in the literature, there remains a gap in other models (8), mainly in developing countries. This raises a question regarding their accuracy and generalizability in different populations, ethnic groups, environmental, and lifestyle factors (9). Previous studies have attempted to address this gap by using surrogate markers such as metabolic syndrome or subclinical atherosclerosis to evaluate the performance of cardiovascular risk models in settings where long-term outcome data are limited, including work conducted in South Asian and African populations (10, 11). It is essential to assess these models' performance in various contexts to ensure appropriate cardiovascular risk assessment and successful preventative measures (12). Previous studies have noted that the Carotid Intima Media Thickness (CIMT) is a marker of subclinical cardiovascular disease (13). In addition, CIMT indicates early structural changes in arterial walls have been widely used as a surrogate Indicator of atherosclerosis before the onset of clinical disease (13). It is also non-invasive and directly measures the increase in the carotid artery's two inner layers (14).

Despite the rising prevalence of CVD in Africa (15, 16), instruments for risk prediction that are specific to the region, and that consider the socioeconomic, lifestyle, and environmental traits of African populations, are still lacking (17). Most available models were developed based on European or North American cohorts, limiting their relevance and applicability in African populations. As a result, the use of non-local models may lead to underestimation or overestimation of CVD risk, potentially compromising prevention and management efforts. This gap often results in risk misclassification, inadequate prevention measures, and missed chances for early intervention. In this context, assessing cross-sectional association, validating, and comparing established CVD risk models among African populations are an important step toward improving risk prediction accuracy (18). Therefore, the present study aims to identify the best model in risk stratification using CIMT among Africans. It is hypothesized that although these models may demonstrate some correlation with CIMT, their overall performance may be suboptimal, underscoring the need for population-specific risk prediction tools.

## 2 Materials and methods

### 2.1 Study design, study population, and sampling

**Figure 1:**
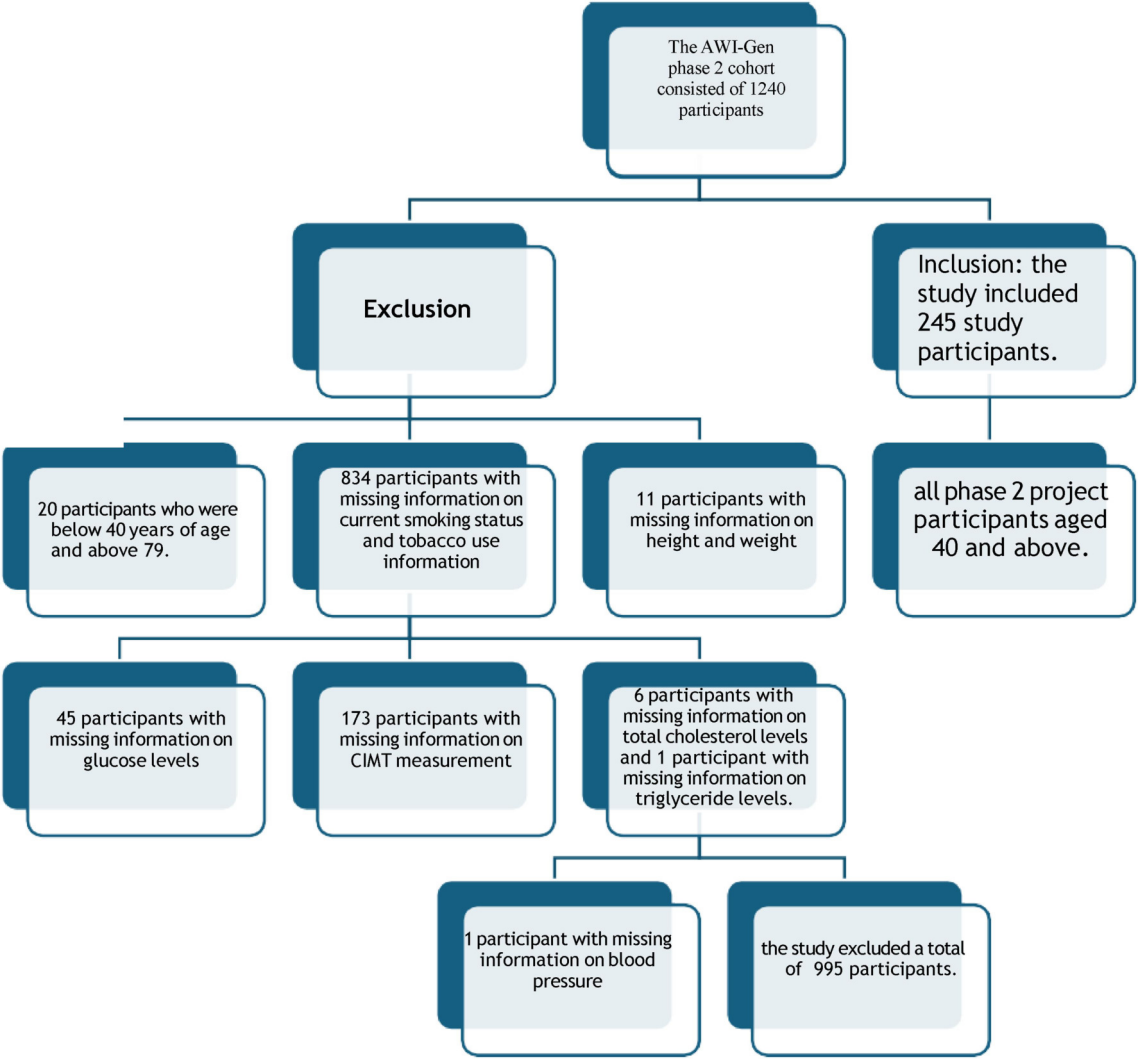
Data screening from AWI-Gen phase 2 cohort.

A cross-sectional analysis of existing data from the Africa Wits-INDEPTH Partnership for Genomic Research(AWI-Gen) phase 2 cohort at the DIMAMO site, with a correlational study design, was adapted in this study. The AWI-Gen phase 2 project selected its participants using a convenience sampling method, and the present study participants' data were selected using the variables needed and an age cut-off of 40 and above. The phase 2 database consisted of 1,240 participants' data collected from 2015 to 2021 in Dikgale, Mamabolo, and Mothiba (DIMAMO) areas. Participants with missing information relevant to the current study and participants with a history of CVD events were excluded. For missing data on relevant variables, I commanded SPSS to exclude cases for missing values. Data screening resulted in 245 participants (see Figure 1). The following variables were extracted from the database: age, height, weight, Systolic Blood Pressure(SBP), Diastolic Blood Pressure (DBP), Total Cholesterol (TC), Triglycerides, High Density Lipoprotein- cholesterol (HDL-C), smoking status, alcohol consumption, averaged (left and right), employment status, and history of CVD events, and glucose levels. All participants provided their consent forms, and the study was approved by the Turfloop Research Ethics Committee (TREC/74/2025: PG). Permission to use AWI-Gen data from the DIMAMO wing was obtained from the DIMAMO principal investigator.

### 2.2 Data collection

The AWI-Gen phase 2 used a validated questionnaire developed by the Research Electronic Data Capture computer database to obtain information on demography, general health, socioeconomic status, and substance use. Height, weight, and blood pressure were measured using a Harpenden digital stadiometer, a digital Physician Large Dial 200 kg capacity, and a digital sphygmomanometer, respectively, while participants were wearing thin and lightweight clothes and on socks. Fasting blood samples were collected by a qualified nurse in accordance with the WHO protocol, and proper procedures and protocols were followed for sample storage and transportation. Serum glucose and lipids were analyzed using a Randox Plus clinical chemistry analyzer. The CIMT was measured using A LOGIQ e ultrasound system with a 2L-RS straight transducer at 1 cm from the bulb of the CCA, procedures will be found in Ali et al. study (19). CIMT was defined as the thickness of the two inner layers of the carotid artery wall (intima-media), and the average CIMT used in this study was calculated from the left CIMT measurements and right CIMT measurements. The Framingham risk model, the ASCVD model, and the ASSIGN risk model were generated manually using Framingham risk charts (20), the ASCVD risk estimator on the American College of Cardiology (21) and ASSIGN(v2.0) (22). The Framingham risk score chart was used to calculate an individual's risk score using participants' data on CVD risk variables manually. The ASCVD risk score calculator was used to compute individual risk scores manually using CVD risk factors. The Assign risk score calculator was used to compute individual risk scores manually using CVD risk factors, and employment status was used for social deprivation.

### 2.3 Diagnostic data analysis

BMI was calculated and categorized according to WHO criteria, with the following categories: normal (BMI ≥ 18.5 kg/m^2^ and <25 kg/m^2^) and obese (BMI ≥ 30 kg/m^2^) (23). Classification of hypertension was guided by the guidelines of the Joint National Committee of Prevention (JNC7), detection, evaluation, and treatment of high blood pressure, where the SBP and/ or DBP of greater than 140 mm Hg and/or 90 mmHg, respectively, were hypertensive (24). Measured total cholesterol level ≥5.0 mmol/L, triglycerides >1.7 mmol/L, and HDL-C < 1.03 mmol/L for men and <1.29 mmol/L for women were considered having elevated total cholesterol, elevated triglycerides, and low HDL-C. Individuals with at least two abnormal lipids mentioned above were regarded as dyslipidemic (25). Classification of diabetes was guided by the standards set by the American Diabetes Association, with individuals having a fasting blood glucose level of ≥7.0 mmol/L being considered diabetic (26). The Framingham risk score (FRS) was determined according to WHO criteria (27): FRS ≤ 10% were low risk, FRS>10% and ≤20% were classified as intermediate risk, and FRS > 20% were classified as high risk (28). The ASCVD risk score was determined according to the American College of Cardiology/American Heart Association, where an ASCVD score <5% was low risk, an ASCVD score ≥5 and <7.5% was intermediate risk, an ASCVD score ≥7.5% and <19,9% high risk, and an ASCVD score ≥20% was very high risk (29). The ASSIGN risk scores were determined according to the Scottish Intercollegiate Guidelines Network (SIGN) and Scottish Government Health Directorates, where a score of ≤19.9% was considered low risk, while a risk score >20% was a high-risk score (30). The CIMT was used as an output variable, classified into normal CIMT and increased CIMT, with normal participants having CIMT < 0,6 mm and high-risk participants having CIMT ≥ 0,6 mm, and this differs by geographic region, age, and sex (31). Other studies use a CIMT cut-off of 0.70 mm to 0.90 mm, but the present study used 0.60 mm as it aims to classify those with sub-clinical atherosclerosis without overt symptoms or clinical events for early detection. Due to the present study sample size CIMT cut off 0.60 mm was partly used to ensure sufficient events for analysis.

### 2.4 Statistical analysis

Data was analysed using the Statistical Package for Social Sciences (SPSS) version 30 software. The variables that are normally distributed were expressed as the mean ± standard deviation, and variables that are not normally distributed were expressed as the median interquartile range (IQR). Chi-square test was used to determine the proportion of categories between participants with normal and increased CIMT. A parametric T-test was used to compare continuous CVD risk variables and CVD risk scores between those with normal and increased CIMT. Linear regression (R statistics) was used to assess the relationship and collinearity of CVD risk models using CIMT. Logistic regression was used to determine the odds of having increased CIMT when the risk scores increase, and the ROC analysis was used to evaluate and compare the CVD risk models' performance using CIMT. A level of significance was set at a *p*-value of equal to or less than 0.05.

## 3 Results

### 3.1 The proportions of CVD risk variables and CVD risk scores among the total population and by participants with normal and increased CIMT. Supplementary material A shows characteristics of participants by gender

Table 1 shows the characteristics of participants for the total population and by risk profile (CIMT). The total number of participants was 245, with 197 participants having variable data on average CIMT, and more participants had increased CIMT (*N* = 139) compared to those with normal CIMT (*N* = 58).The mean age of the total population was 59.22 ± 8.14, and no statistical difference between those with normal and increased CIMT.

**Table 1:** Characteristics of participants by risk profile.

Variables	Risk profile		*P*-value
	Normal CIMT (*N* = 58)	Increased CIMT (*N* = 139)	
Female	49 (84.5)	105 (75.5)	0.143
Age (years)	58.19 ± 8.60	60.15 ± 8.02	
BMI (kg/m^2^)	29.96 (24.29–38.18)	30.84 (25.56–36.49)	0.837
Obesity N(%)	29 (50)	77 (55.4)	0.825
SBP (mmHg)	130.00 (118.00–146.00)	127.00 (116.00–140.00)	0.667
DBP (mmHg)	78.50 (72.00–86.24)	78.00 (72.00–84.00)	0.761
Hypertension N(%)	23 (39.7)	38 (27.3)	0.264
TC (mmol/L)	4.77 ± 1.22	4.65 ± 1.00	0.579
Proportion of increased TC N(%)	15 (25.9)	37 (27)	0.497
Trig (mmol/L)	1.31 (0.93–1.76)	1.22 (0.91–1.75)	0.766
Proportion of increased trig N(%)	14 (25.9)	37 (27)	0.879
HDL-C (mmol/L)	1.19 (0.93–1.35)	1.13 (0.95–1.31)	0.423
Proportion of low HDL-C N (%)	18 (31)	61 (43.9)	0.118
Dyslipidemia N(%)	23 (39.7)	68 (48.9)	0.211
Glucose level (Mmol/L)	5.58 ± 1.05	6.44 ± 2.97	0.038
Diabetes mellitus N(%)	24 (12.6)	7 (13)	0.185
Tobacco use N(%)	8 (13.8)	17 (12.24)	0.657
Alcohol consumption N(%)	14 (24.1)	35 (25.5)	0.833
FRS (%)	7.30 (5.50–15.90)	8.60 (6.30–15.60)	0.413
Proportion intermediate FRS N(%)	11 (20.4)	37 (27.0)	0.341
Proportion of high-risk FRS N(%)	8 (14.8)	23 (16.8)	0.739
ASCVD scores (%)	5.00 (2.00–10.00)	5.00 (3.00–9.75)	0.573
Proportion of intermediate ASCVD score N(%)	9 (16.7)	25 (18.2)	0.932
Proportion of high ASCVD score N(%)	13 (24.1)	27 (19.7)	0.369
Proportion of very high ASCVD score N(%)	3 (5.6)	11 (8.0)	0.620
Assign risk scores (%)	3.65 (1.58–3.60)	4.10 (2.53–6.58)	0.346
Proportion of high ASSIGN risk score N(%)	1(1.9)	7(5.1)	0.308

SBP, Systolic Blood Pressure; DBP, Diastolic Blood Pressure; TC, Total Cholesterol; trig, triglycerides; HDL-C, High Density Lipoprotein-Cholesterol; FRS, Framingham Risk Score; ASCVD, Atherosclerotic Cardiovascular Disease, *p* < 0,050 is significant.

The BMI (IQR) was also not statistically different between participants with normal CIMT and those with increased CIMT. The systolic blood pressure, diastolic blood pressure, HDL-C, triglycerides, and total cholesterol levels. The FRS, ASCVD risk score, and ASSIGN risk score were statistically not different among individuals with normal and increased CIMT. The glucose levels were significantly higher among participants with increased CIMT compared to those with normal CIMT. The proportion of intermediate and high-risk FRS among the total population was 25,7% and 14,7% respectively, and there was no statistical difference among participants with normal and increased CIMT. The proportion of ASCVD intermediate risk, high risk, and very high risk among the total population was 19,2%,19,2% and 6,1% respectively. The proportion of intermediate risk, high-risk, and very high-risk ASCVD in participants with normal CIMT was 24.8%, 28.2% and 9.7% respectively, 20%, 18.3%, and 6.7% in participants with increased CIMT, respectively, with no statistical significance. The proportion of participants with an ASSIGN high-risk score among the total population was 3,3% and there was no significant difference between participants with normal CIMT and those with increased CIMT.

### 3.2 Determination of the relationship between CVD risk scores from each CVD risk model and CIMT

In the present study, the scatter plot shows that there is a positive correlation between FRS risk score, ASCVD risk score, ASSIGN risk score, and CIMT. The analysis for the FRS shows a very weak positive correlation with CIMT. The ASCVD and ASSIGN risk models also showed weak positive correlations with CIMT. The scatter plots showing the correlation will be found in Supplementary material B.

### 3.3 Determination of the odds ratio of having increased CIMT

Table 2 shows the odds ratios of CVD risk models and CIMT. The Framingham risk score, the ASCVD risk score, and the Assign risk score were used to determine the odds of having increased CIMT without unadjusted. The multivariate logistic regression results indicate that there is no significant association between the CVD risk models and an increased CIMT. Table 3 shows that even after adjusting for age and gender, there was no significant association among the CVD risk models.

**Table 2:** Comparison of CVD risk models odds using the multivariate logistic regression model, unadjusted.

Variables	CIMT	Odds ratio	95% CI	*P*-value
Framingham risk score	Normal CIMT	1		
	Increased CIMT	0.980	0.898–1.069	0.649
ASCVD risk score	Normal CIMT	1		
	Increased CIMT	0.966	0.845–1.105	0.614
ASSIGN risk model	Normal CIMT	1		
	Increased CIMT	1.103	0.915–1.330	0.302

**Table 3:** Comparison of CVD risk models odds using the multivariate logistic regression model adjusted for age and gender.

Variables	CIMT	Odds ratio	95% CI	*P*-value
Framingham risk score	Normal CIMT	1		
	Increased CIMT	0.958	0.84–1.051	0.364
ASCVD risk score	Normal CIMT	1		
	Increased CIMT	0.876	0.742–1.033	0.116
ASSIGN risk model	Normal CIMT	1		
	Increased CIMT	1.182	0.952–1.468	0.129

### 3.4 Comparison of receiver operating characteristics (ROC) to determine the accuracy of models

**Figure 2:**
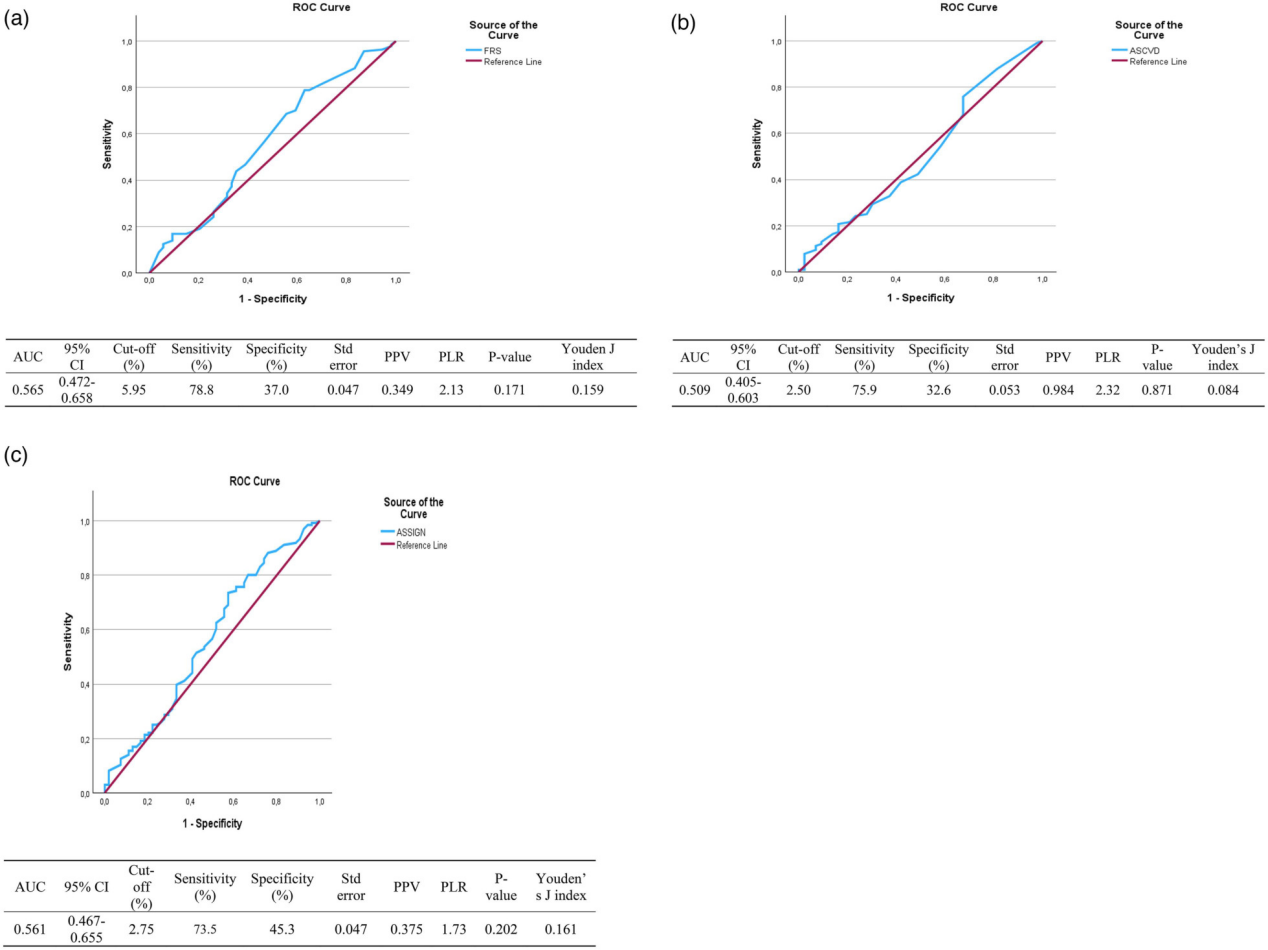
Coordinates for ROC for FRS: (a) receiver operative characteristics for the framingham risk score (%) as a predictor of CIMT (mm). Coordinates for ROC for ASCVD: (b) Receiver operative characteristics for the ASCVD risk score (%) as a predictor of CIMT (mm). Coordinates for ROC for ASSIGN: (c) Receiver operative characteristics for the ASSIGN risk score (%) as a predictor of CIMT (mm).

Figure 2 shows receiver operating characteristics (ROC) for CVD risk models using CIMT. In the present results, the maximum Youden index was recorded together with its corresponding cut-off, sensitivity, specificity, and *p*-value. These ROC cut-offs for the CVD risk scores indicate that for this study population, the models perform satisfactorily when they need to use the reference category of 0.60 mm. The ROC used a non-parametric approach to calculate the AUC confidence intervals.

Among the total population, the FRS cut-off value of 5.95% gave the highest sensitivity of 78.8%, the specificity of 37% and an area under the curve (AUC) of 0,565. The Youden index (YI), 95% confidence interval (CI), positive likelihood ratio (PLR), positive predictive value (PPV), and the *p* value were 0.159, 0.472–0.658,2.13,0.770, *p* = 0.171, respectively. The results show that the FRS is not good at identifying people with high CIMT from those with normal CIMT (Table 4).

**Table 4:** ROC analysis between CVD risk models and CIMT among the total study population.

Variables (%)	(AUC)	95% CI	Cut-off	Sensitivity (%)	Specificity (%)	Std error	PPV	PLR	Youden index	*P*-value
Framingham risk model	0.565	0.472–0.658	5.95	78.8	37.0	0.047	0.349	2.13	0.159	0.171
ASCVD risk model	0.509	0.405–0.613	2.50	75.9	32.6	0.053	0.984	2.32	0.084	0.871
ASSIGN risk model	0.561	0.467–0.655	2.75	73.5	45.3	0.047	0.375	1.73	0.161	0.202

Among the total population, the ASCVD risk score cut-off value of 2,50% gave the highest sensitivity of 75,9%, the specificity of 32,6% and an area under the curve (AUC) of 0,509. The Youden index (YI), 95% confidence interval (CI), positive likelihood ratio (PLR), positive predictive value (PPV), and the *p* value were 0.084, 0.405–0.613,2.32,0.984, *p* = 0.871, respectively. The results show that the ASCVD risk score is not good at identifying people with high CIMT compared to those with normal CIMT (Table 4).

Among the total population, the ASSIGN risk score cut-off value of 2,75% gave the highest sensitivity of 73,5%, the specificity of 45,3% and an area under the curve (AUC) of 0,561. The Youden index (YI), 95% confidence interval (CI), positive likelihood ratio (PLR), positive predictive value (PPV), and the *p* value were 0.161, 0.467–0.655,1.73,0.467, *p* = 0.202, respectively. The results show that the ASSIGN risk score is not effective at identifying individuals with high CIMT compared to those with normal CIMT (Table 4). The 0.60 mm threshold showed greater predictive values and stronger correlations than when a cut-off of 0.70 mm.

Figures 2a–c show Receiver Operating Characteristics (ROC) for the Framingham risk model, the ASCVD risk model, and the ASSIGN risk model, respectively, with CIMT.

## 4 Discussion

Fewer studies have associated the CVD risk scores with CIMT among Africans in South Africa, which makes it difficult to compare the results of the present study with similar studies. To strengthen the contextual interpretation, it is important to acknowledge that the limited availability of African-specific evidence restricts direct benchmarking of these findings. In the present study, the proportion of individuals with an intermediate Framingham Risk Score (FRS) was 25.7% of the total population, which is lower than the 38.7% reported by Nyirenda et al. (32) among South Africans. This difference may be due to the difference in the study setting and population, such as the study area, number of participants, age distribution, and lifestyle factors in cardiovascular risk profiles. These contextual differences, particularly the rural nature of the present study vs. urban or semi-urban settings in prior studies, may partially explain the lower observed proportions. Lifestyle factors are more common in urban areas than in rural areas (33). Nyirenda et al. (32) conducted their study in the semi-urban and urban areas of Durban in Kwa-Zulu-Natal with study participants aged 50 years and above. Similarly, a study by Nyirenda et al. reported a prevalence of a high FRS was 28%, which is higher than 14,7% reported in the present study (32).

According to the author's knowledge, this is the first study to be conducted on ASCVD among Africans, highlighting a major evidence gap in ASCVD risk profiling within the African populations. In the present study, the proportion of intermediate ASCVD score and high-risk ACVD risk score was reported to be 19,2% and 25,9% respectively, among the total population. The present study reported a lower proportion compared to the study by Fernández-Friera et al. (34), conducted among white population aged 40–54 years old, Fernández-Friera et al. (34), which reported that intermediate and high-risk ASCVD scores were 24% and 37% respectively. The difference may be that CVDs are more common among whites (35).

According to the author's knowledge, there are no studies conducted on ASSIGN among Africans, hence the comparison to previous studies was also challenging. In the present study, the proportion of high ASSIGN risk score was reported to be 3,3% among the total population. This is in contrast with Van Staa et al.'s study (36), as they reported a higher prevalence of the ASSIGN high-risk score. Van Staa et al. (36) conducted their study in a multi-ethnic population of 1,853,186 participants aged 35–74 years, with only 1,3% of black men and 1,6% of black women. CVD risk and prevalence is different among different racial groups (35).

The current study found no significant difference in the proportion of intermediate FRS and high FRS between normal CIMT and increased CIMT participants. This contrasts with a study by Karim et al. (37), findings as they reported that participants with increased CIMT had significantly higher values than participants with normal CIMT. A study by Karim et al. (36) also reported that 37% of high FRS in participants with increased CIMT was significantly higher than that of normal CIMT participants. Karim et al. (37) conducted their study in a multiethnic population with 498 participants which comprised 14% black participants, while the majority of the population were white participants. These population differences, particularly racial composition, may contribute to divergent findings due to variation in CVD risk distributions (35).

The present study showed no statistical difference in the proportion of intermediate ASCVD, high ASCVD, and very high ASCVD between normal CIMT and increased CIMT participants. This contrasts with a study by Nonterah et al. (18), as they reported that participants with increased CIMT have significantly higher high-ASCVD scores compared to normal CIMT participants. A study by Nonterah et al. (18) used a CIMT cut-off of >0,9 mm, whereas the present study used >0.6 mm, indicating a difference in group classification, which may explain the variation in results. Although higher thresholds (≥0.8–0.9 mm) are commonly used to define more advanced subclinical atherosclerosis, normative population data indicate that mean CIMT values between approximately 0.6 and 0.7 mm represent the upper range of normal in healthy adults, and median CIMT values in several African cohorts are often below 0.7 mm. Therefore, the lower cut-off applied in this study was intended to capture early arterial wall thickening rather than establish atherosclerosis, but it may have increased prevalence estimates and reduced between-group contrast**.**

The present study showed that there was no statistical difference between participants with normal CIMT and participants with increased CIMT in the proportion of high ASSIGN risk scores. This contradicts with a study by Hosein et al. (38), as they reported that participants with increased CIMT have a significantly higher proportion of high-ASSIGN risk score. The contradiction in results may also be due to the differences in the racial groups used, as a study by Hosein et al. (38) was among multi-racial individuals, while the present study was done on the rural black population.

In the present study, the Framingham risk model showed a positive correlation with CIMT. This is in alignment with a study by Mitu et al. (39), as they reported that the Framingham risk score has a positive correlation with CIMT and a significant association.

In the present study, the ASCVD risk model showed a positive correlation with CIMT. This aligns with a study by Li et al. (40), as they reported a positive correlation between ASCVD and CIMT. In the present study, the ASSIGN risk model showed a positive correlation with CIMT. This agrees with a study by De La Iglesia et al. (41), as they reported a positive relationship between the ASSIGN model and CIMT in the UK participants. These consistent correlations across different models suggest that, although estimation accuracy is limited, the risk scores retain some relevance in estimating vascular burden, with a lack of discriminatory and clinical utility, as they have wider confidence intervals.

Data supporting the present study's ROC is limited, as this study is among the few studies that determine the CVD risk models' estimation accuracy and discriminative ability. Several factors may explain the poor discriminative performance of the cardiovascular risk models observed in this study. First, the relatively small sample size may have limited statistical power and contributed to wide confidence intervals around the AUC estimates, thereby reducing the precision of the ROC analysis. Second, outcome misclassification may have occurred due to the use of a lower CIMT cut-off (≥0.6 mm), which may have inflated the prevalence of increased CIMT and reduced contrast between comparison groups, ultimately attenuating model discrimination. Third, differences in cardiovascular risk factor distributions and competing risk structures in rural African populations, compared to the predominantly European and North American populations in which the Framingham, ASCVD, and ASSIGN models were developed, may further limit model performance. These populations differ in baseline risk profiles, exposure patterns, and non-cardiovascular competing causes of morbidity and mortality, which may weaken the relationship between conventional risk scores and subclinical atherosclerosis.

The present study's ROC analysis of FRS and ASSIGN risk score shows a low AUC when compared to a study by Bhuiyan et al. (8), as reported higher values (8). Bhuiyan et al. (8) used a larger sample size compared to the present study, with a multi-ethnic population, and they distinguished CVD events, while the present study used CIMT. The present study showed poor model performance with wider AUC confidence intervals, and these might be due to the sample size used; hence, it cannot accurately distinguish sub-clinical CVD. Further studies are needed on these CVD risk models in discriminating established CVD.

When compared with other African cohorts, the findings of this study demonstrate both consistencies and important contextual differences in cardiovascular risk profiling. Similar to reports by Nyirenda et al. (32) and Nonterah et al. (18), this study observed modest associations between cardiovascular risk scores and subclinical atherosclerosis, although the magnitude of risk differed across populations. While Nyirenda et al. reported higher proportions of intermediate and high Framingham risk in semi-urban South African populations, and Nonterah et al. (18), observed stronger associations between ASCVD scores and CIMT in West African cohorts, the present study showed generally lower risk estimates and weaker discrimination. These differences likely reflect variation in demographic composition, rural, urban context, cardiovascular risk factor distribution, and lifestyle patterns across African settings. Collectively, these findings highlight substantial heterogeneity in CVD risk expression across African populations and underscore the importance of developing and validating context-specific risk prediction tools rather than relying solely on models derived from non-African or urban cohorts. In addition, the characteristics of the AWI-Gen Phase 2 cohort should be considered when interpreting model discrimination. The dataset comprises community-based, apparently healthy adults with a relatively low prevalence of overt cardiovascular disease. In such populations, limited variation in clinical risk status and a low burden of advanced disease may inherently constrain the discriminative capacity of cardiovascular risk models, resulting in lower AUC estimates even when models are well calibrated for predicting long-term clinical events. Consequently, the modest discrimination observed in this study should not be interpreted as definitive evidence of model failure, but rather as a reflection of the underlying population risk profile and the use of a subclinical surrogate outcome.

The findings of this study have important implications for cardiovascular risk assessment in rural African populations. First, the limited performance of commonly used CVD risk models such as FRS, ASCVD, and ASSIGN in distinguishing increased CIMT suggests these tools may not be fully suitable for evaluating subclinical atherosclerosis in rural Black South Africans. Second, the correlations observed between risk scores and CIMT, despite their poor association accuracy, highlight the need for locally validated or population-specific risk prediction models. Third, the differences identified between this study and previous research in predominantly white or multi-ethnic populations emphasize how demographic, lifestyle, and genetic factors influence CVD risk profiling. Finally, the study adds to the limited evidence on the usefulness of CVD risk scores in African contexts and underscores the necessity for future research incorporating longitudinal data, larger sample sizes, and additional biomarkers to enhance early detection and prevention methods.

### 4.1 Limitations and strengths

The findings of this study did not associate with gender as the sample size for male participants with increased CIMT was lower (*N* = 34), and the study used retrospective data that was sampled using convenience sampling, which limits generalization. The large proportion of excluded participants due to missing data resulted in a significant limitation for comparability to previous studies, which might have affected the discriminative capacity of CVD risk models due to the low prevalence of CVD risk factors, and has affected the generalizability of the present study results. The present study is the first of its kind, and as a result, data supporting the CIMT cut-off and the present study's findings are limited. The study used retrospective data, which may be subject to bias and inaccuracy due to missing values of CIMT levels in other participants. Additionally, the use of a lower CIMT threshold (≥0.6 mm) may have inflated the prevalence of increased CIMT and reduced between-group variability, which could have influenced model performance estimates. The strength of the study is that the use of quantitative analysis allows for the identification of significant associations between CVD risk models and CIMT. Retrospective design reduces cost and time in steps such as data collection.

## 5 Conclusion

There was no difference in the proportion of participants with risk scores using each model between participants with normal and increased CIMT. The multivariate regression showed that these CVD risk scores cannot discriminate against increased CIMT. The ROC showed that these CVD risk scores have a poor discriminative ability to correctly classify participants at risk (having increased CIMT) from those who are normal among residents of DIMAMO. Overall, in this small, highly selected rural sample, discrimination for increased CIMT was limited. In light of the findings of the present study. Further studies with a larger sample size are recommended, and further association by gender for a deeper understanding and analysis. Similar studies should be conducted among Africans in urban and rural areas to validate the present study results.

## Data Availability

The datasets presented in this article are not readily available due to ethical restrictions and the confidentiality of the database from which the data was extracted. Requests to access the datasets should be directed to the corresponding author(s).
