# Supplementary material for: Comparison of framingham risk model, atherosclerotic cardiovascular disease risk model, and assign risk model in detecting sub-clinical atherosclerosis among DIMAMO residents, Limpopo province, South Africa
Source: Front Cardiovasc Med. 2026 Feb 19;13:1726722. doi: 10.3389/fcvm.2026.1726722 (PMC12960639; doi:10.3389/fcvm.2026.1726722)
Supplement: Supplementary file 1 [file Datasheet1.docx]

Supplementary Material A

*Characteristics of participants by gender*

| Variables | Total | Females=191 | Males=54 | p-value |
| --- | --- | --- | --- | --- |
| Age (years) | 59.22±8.143 | 59.03±8.184 | 59.59±8.032 | 0.493 |
| BMI (kg/m^2^) | 30.78(25.09-37.44) | 33.14(28.00-38.81) | 24.38(20.19-29.05) | <0.001 |
| Obesity N (%) | 133(54.3) | 122(63.9) | 11(20.4) | <0.001 |
| SBP (mmHg) | 128(116.00-142.00) | 128.00(118.00-142.00) | 124.00(113.75-139.75) | 0.664 |
| DBP (mmHg) | 78.00(72.00-84.00) | 78.00(71.00-85.00) | 78.00(72.75-82.00) | 0.822 |
| Hypertension N(%) | 74(30.2) | 60(31.4) | 14(25.9) | 0.438 |
| TC (mmol/l) | 4.69±1.07 | 4.83±1.06 | 4.14±1.06 | <0.001 |
| Proportion of increased TC N(%) | 79(32.2) | 67(35.1) | 12(22.2) | 0.074 |
| Trig (mmol/l) | 1.35±0.90 | 1.37±0.58 | 1.28±0.65 | 0.309 |
| Proportion of increased trig N(%) | 63(25.7) | 51(26.7) | 12(22.2) | 0.506 |
| HDL-C (mmol/l) | 1.18± 0.32 | 1.16±0.29 | 1.15±0.41 | 0.346 |
| Proportion of low HDL-C N (%) | 93(38.0) | 55(28.8) | 38(70.4) | <0.001 |
| Dyslipidemia N(%) | 111(45.3) | 72(37.7) | 12(22.2) | <0.001 |
| Glucose level (mmol/l) | 6.04±2.39 | 6.10±2.49 | 5.84±2.01 | 0.484 |
| Diabetes mellitus N(%) | 31(12.7) | 24(12.6) | 7(13.0) | 0.938 |
| Tobacco use N(%) | 32(13.1) | 5(2.6) | 27(50.0) | <0.001 |
| Alcohol consumption | 64(26.1) | 30(15.7) | 34(63.0) | <0.001 |
| FRS(%) | 8.60(5.30-13.70) | 7.30(4.50-11.70) | 15.60(9.40-30.00) | <0.001 |
| Proportion of intermediate FRS N(%) | 63(25.7) | 47(24.6) | 16(29.6) | 0.456 |
| Proportion of high FRS N(%) | 36(14.7) | 13(6.8) | 23(42.6) | <0.001 |
| ASCVD risk score (%) | 5.00(2.00-9.00) | 4.00(2.00-7.00) | 13.00(5.00-21.50) | <0.001 |
| Proportion of intermediate ASCVD score N(%) | 47(19.2) | 43(22.5) | 4(7.4) | 0.051 |
| Proportion of high ASCVD score N(%) | 47(19.2) | 32(16.8) | 15(27.8) | 0.005 |
| Proportion of very-high ASCVD score N(%) | 15(6.1) | 4(2.1) | 11(20.4) | <0.001 |
| ASSIGN risk score (%) | 3.80(2.03-5.80) | 3.40(1.80-5.30) | 5.60(3.65-10.50) | <0.001 |
| Proportion of high ASSIGN risk score N(%) | 8(3.3) | 3(1.6) | 5(9.3) | 0.004 |
| CIMT (mm) | 0.67±0.12 | 0.67±0.13 | 0.47±0.50 | 0.799 |
| Proportion of increased CIMT N(%) | 139(56.7) | 105(55.5) | 34(63.0) | 0.166 |

*SBP- Systolic Blood Pressure, DBP-Diastolic Blood Pressure, TC-Total Cholesterol, trig- triglycerides, HDL-C: High Density Lipoprotein-Cholesterol, FRS-Framingham Risk Score, ASCVD-Atherosclerotic Cardiovascular Disease, p<0,050 is significant.

Supplementary Material B


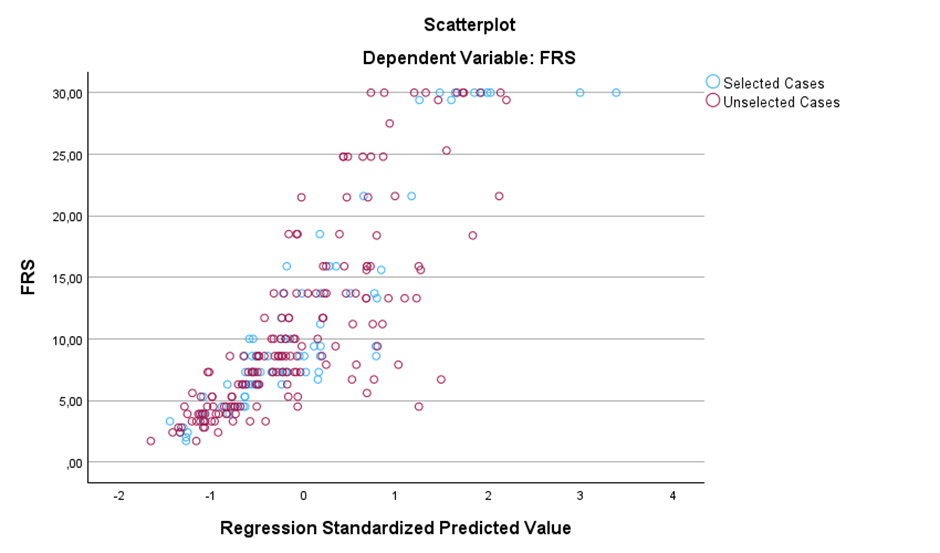


| Variable | Coefficient correlation | 95% CI | P-value |
| --- | --- | --- | --- |
| FRS | 0.016 | -0.128-0.160 | 0.821 |

(a) The scatterplot of the Framingham risk score (%) as a predictor for the CIMT(mm).


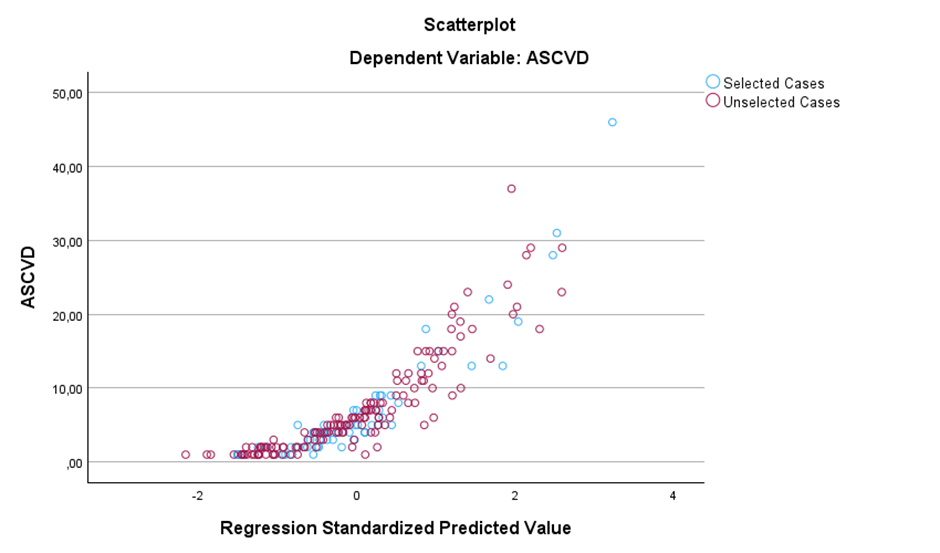


| Variable | Coefficient correlation | 95% CI | P-value |
| --- | --- | --- | --- |
| ASCVD | -0.104 | -0.258-0.055 | 0.185 |

(b) The scatterplot of the ASCVD risk score (%) as the predictor of CIMT(mm).


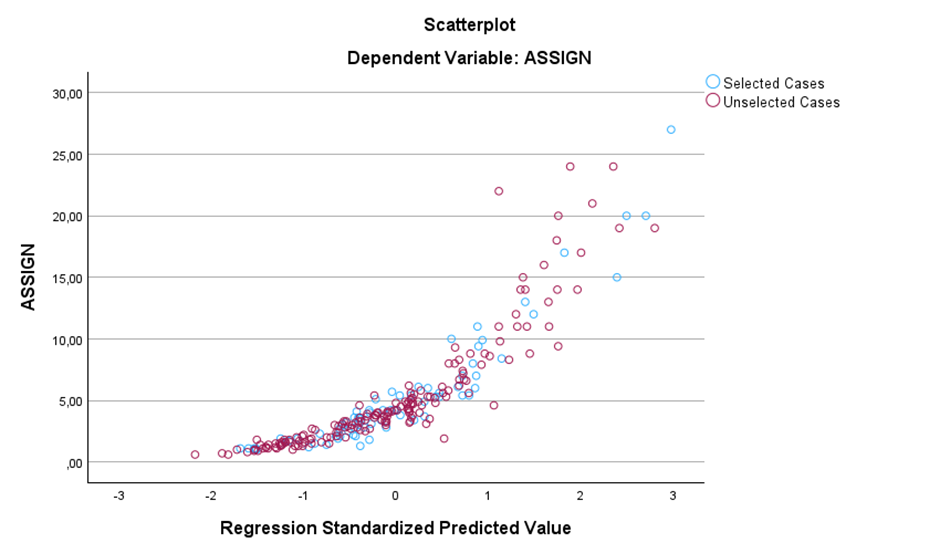


| Variable | Coefficient correlation | 95% CI | P-value |
| --- | --- | --- | --- |
| ASCVD | 0.014 | -0.126-0.154 | 0.846 |

(c ) The scatterplot of the ASSIGN risk score (%) as a predictor of CIMT (mm).

|  |  |
| --- | --- |

**Figure 3.** Scatterplots of CVD risk scores predicted using CIMT.
